# Neurocognitive effects of proanthocyanidin in Alzheimer's disease: a systematic review of preclinical evidence

**DOI:** 10.1590/1414-431X2024e13587

**Published:** 2024-11-04

**Authors:** A. Reshma, A. Subramanian, V. Kumarasamy, T. Tamilanban, M. Sekar, S.H. Gan, V. Subramaniyan, L.S. Wong, N.N.I.M. Rani, Y.S. Wu

**Affiliations:** 1 Department of Pharmacology, SRM College of Pharmacy, SRM Institute of Science and Technology, Kattankulathur, Chengalpattu, Tamilnadu, India; 2 Department of Parasitology & Medical Entomology, Faculty of Medicine, Universiti Kebangsaan Malaysia, Jalan Yaacob Latif, Kuala Lumpur, Malaysia; 3 Department of Occupational Safety and Health, Faculty of Public Health, Universitas Airlangga, Surabaya, Indonesia; 4 Faculty of Health and Life Sciences, INTI International University, Nilai, Malaysia; 5 Department of Pharmacology, Faculty of Medicine, MAHSA University, Bandar Saujana Putra, Selangor, Malaysia; 6 School of Pharmacy, Monash University Malaysia, Bandar Sunway, Selangor, Malaysia; 7 Department of Medical Sciences, School of Medical and Life Sciences, Sunway University, Bandar Sunway, Selangor, Malaysia; 8 Faculty of Pharmacy and Health Sciences, Royal College of Medicine Perak, Universiti Kuala Lumpur, Perak, Malaysia; 9 Sunway Microbiome Centre & Department of Biological Sciences, School of Medical and Life Sciences, Sunway University, Subang Jaya, Selangor, Malaysia

**Keywords:** Alzheimer's disease, Cognition, Dementia, Flavonoids, Proanthocyanidin, Human health

## Abstract

Cognitive disorders and dementia largely influence individual independence and orientation. Based on the Alzheimer's Disease International (ADI) estimation, approximately 75% of individuals with dementia are undiagnosed. In fact, in some low- and middle-income countries, the percentage is as high as 90%. In this systematic review, which is based on PRISMA guidelines, we aim to identify the mechanism of action of proanthocyanidin. Finding a natural product alternative as a potential nootropic can help increase the number of armamentariums against dementia and other cognitive impairments. In this preclinical research, we determined the effect of proanthocyanidins on Alzheimer's disease (AD) by searching electronic bibliographic databases like Scopus, Proquest, ScienceDirect, PubMed, and Google. There was no imposed time limit. However, the search was limited to only English articles. The review protocol is registered on PROSPERO as CRD42022356301. A population, intervention, control, and outcomes (PICO) technique was utilized for report inclusion, and all reports were assessed for risk of bias by using the SYRCLE's RoB tool. The article's bibliographic information, induction model, type of proanthocyanidins, animal strain/weight/age, and outcome measurements were acquired from ten papers and are reported here. Further analysis was validated and determined for the review. The included studies met the review's inclusion criteria and suggested that proanthocyanidins have a neurocognitive effect against AD. Additionally, the effectiveness of proanthocyanidins in reducing oxidative stress, acetylcholinesterase activity, amyloid beta, its efficacy in alleviating superoxide dismutase, cognitive properties, and in facilitating cholinergic transmission in various models of AD has been collectively observed in ten studies.

## Introduction

**Figure 1:**
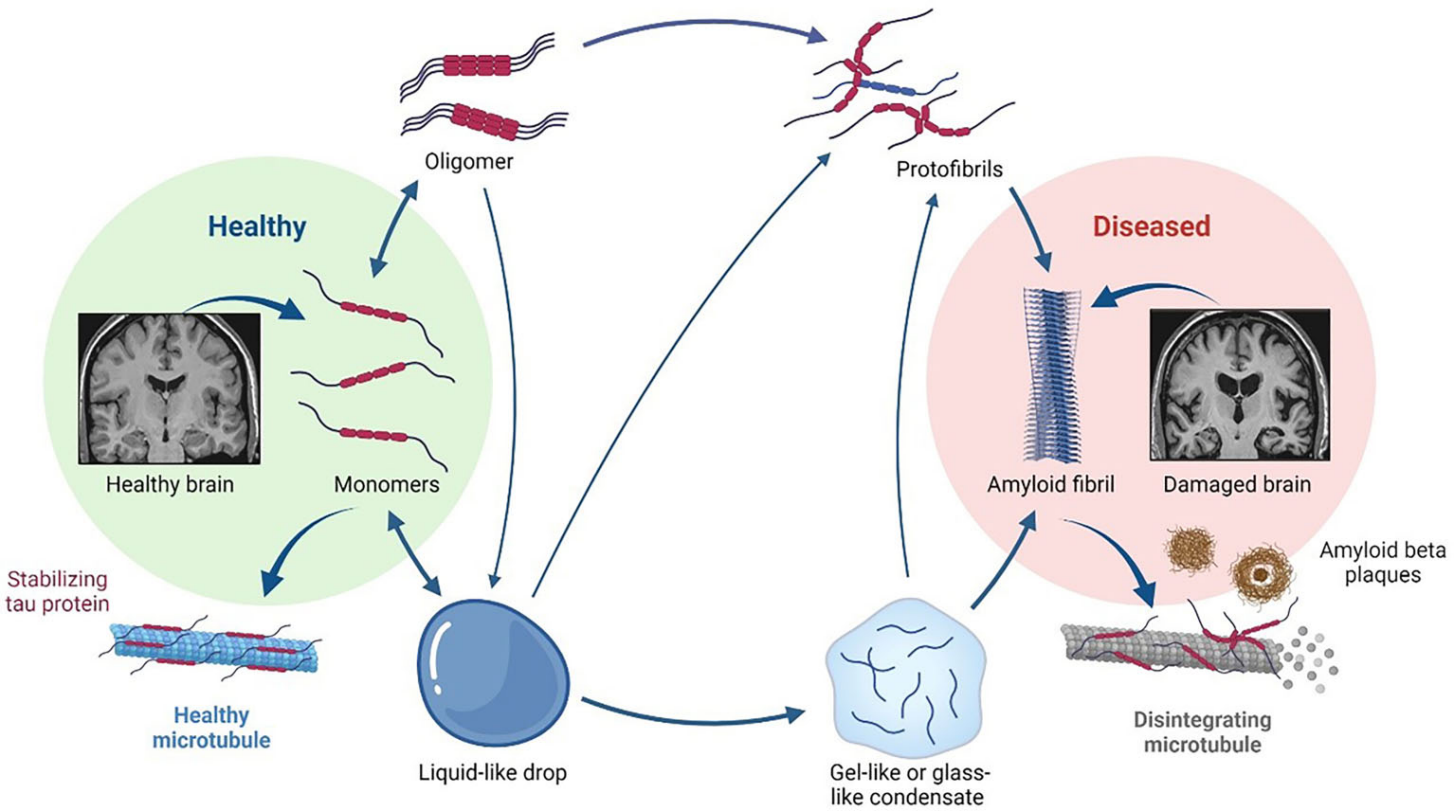
Mechanism of neurodegeneration in Alzheimer's disease. In the healthy brain, there is no formation of beta-amyloid plaques and neurofibrillary tangles, whereas in the diseased brain, insoluble deposits of beta-amyloid peptide occur resulting in hyperphosphorylation of tau proteins, which are the pathological hallmarks of Alzheimer's disease. This results in gradual death of neuronal cells in particular regions of the brain.

The World Health Organization (WHO) reports that there are approximately 10 million new cases of dementia every year, impacting 55 million people globally. In low- and middle-income nations, Alzheimer's disease (AD) accounts for almost 60% of all dementia cases. The prevalence is anticipated to increase to 78 million in 2030 and 139 million in 2050. AD, which accounts for 60-70% of dementia cases, is the most common type (1). AD is a neurodegenerative condition that worsens over time (Figure 1). It is characterized by the presence of neurofibrillary tangles (NFTs) inside neurons and senile plaques composed of the amyloid-beta (Aβ) peptide, neuritic plaques, and Aβ aggregates that form outside neuronal cells, as well as tau protein hyperphosphorylation (2). Amyloid plaques disrupt the synaptic connections between neurons, resulting in neurodegeneration. Tau is a microtubule that controls the movement of nutrients inside neurons and stabilizes them internally. NFTs are formed when tau proteins aggregate abnormally to form paired helical filaments that obstruct the transport of nutrients, ultimately causing neurotoxicity (3,4).

Current treatment includes the use of cholinesterase inhibitors for AD dementia patients at any stage, while memantine is an option for those with moderate-to-severe AD dementia. When taken at the right time during the disease, these drugs improve both the patient's and caregiver's quality of life, although they do not impact disease duration nor the rate of decline (5). Recently, the Food and Drug Administration (FDA, USA) approved Lecanemab, a monoclonal antibody medication that can be used to treat AD. However, it is quite expensive, and may cause side effects such as dizziness, headache, and confusion, yet it remains a gold mine (6-8). Hence, the search for a potential compound with lesser cost and adverse effects remains in high demand.

Sufficient knowledge is currently available about AD progression, from the pathophysiology, biomarker, and clinical perspectives, to recognize AD as a continuum, i.e., a progression of pathophysiological changes that lead to clinically evident disease and a gradual deterioration of cognitive and functional capacities. Nevertheless, there are no clear distinctions between the various clinical stages (9).

**Figure 2:**
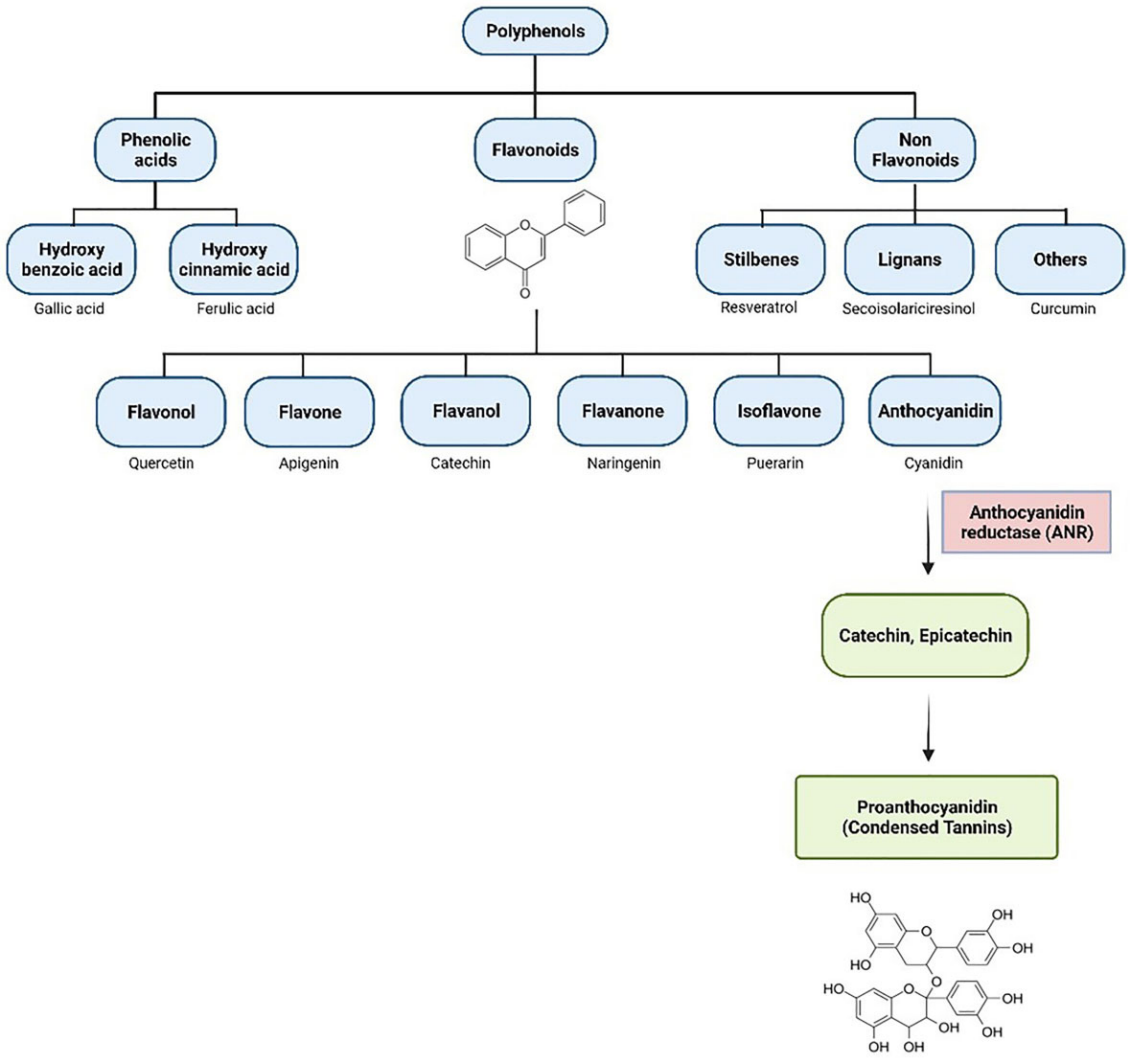
Classes of polyphenols and the bio-synthesis pathway of proanthocyanidin.

Flavonoids are a class of polyphenolic substances confirmed to be beneficial to human health (10-12). Free radical scavenging, metal chelation, and the control of enzyme activity are just several theories behind how polyphenols benefit the brain (13-15). Some flavonoids polymerize to produce tannins, such as flavan-3-ols, catechin, and epicatechin. Tannins are secondary metabolites in plants that are either hydrolyzed or condensed; the latter is also termed proanthocyanidins (16). Proanthocyanidin, present in the flowers, fruits, nuts, and seeds of various plants, protects against biotic and abiotic stresses. They defend the plants from predators due to their astringent nature. The flavonoid biosynthetic pathway yields both oligomeric and polymeric metabolites, with epicatechin and catechin being two of the proanthocyanidins*'* building blocks (depicted in Figure 2) (17). The best sources of *proanthocyanidins* are berries and fruits. Some edible berries with a high concentration of *proanthocyanidins* are lingonberries, cranberries, black elderberries, black chokeberries, black currants, and blueberries (18).

The most prevalent plant-derived polyphenols are members of the second class, which consists of condensed tannins, also known as proanthocyanidins. They are also known as oligomeric proanthocyanidins (OPCs) and are oligomers of flavan-3-ol (catechin monomers) and/or flavan-3,4-diol, typically linked by C-C (4-8 or 6-8) and occasionally by C-O-C bonds with a wide structural diversity. These compounds are not easily hydrolyzed; they decompose under acidic alcoholic conditions to produce phlobaphenes, which are red pigments. However, the chemistry of proanthocyanidin remains only partially understood (19).

Despite being abundant in the human diet (estimated daily intake; 0.1-0.5 g), tannins gain little attention due to their polymeric existence and structural complexity (20). Proanthocyanidins and their monomers have gained a lot of attention recently because of their potential health benefits, which include anti-cancer, immunomodulatory, antioxidant, cardioprotective, anti-inflammatory, and antithrombotic activities (21-24). Nevertheless, due to their vast potential, more information about their pharmacological and toxicological behavior is required to harness their full potential. Extensive biotransformation resulting in extremely low blood and tissue concentrations of unaltered polyphenols, rapid excretion, and relatively poor intestinal absorption are other challenges since they confer further lack of direct antioxidant or other significant systemic effects, particularly in the brain (25-27).

**Figure 3:**
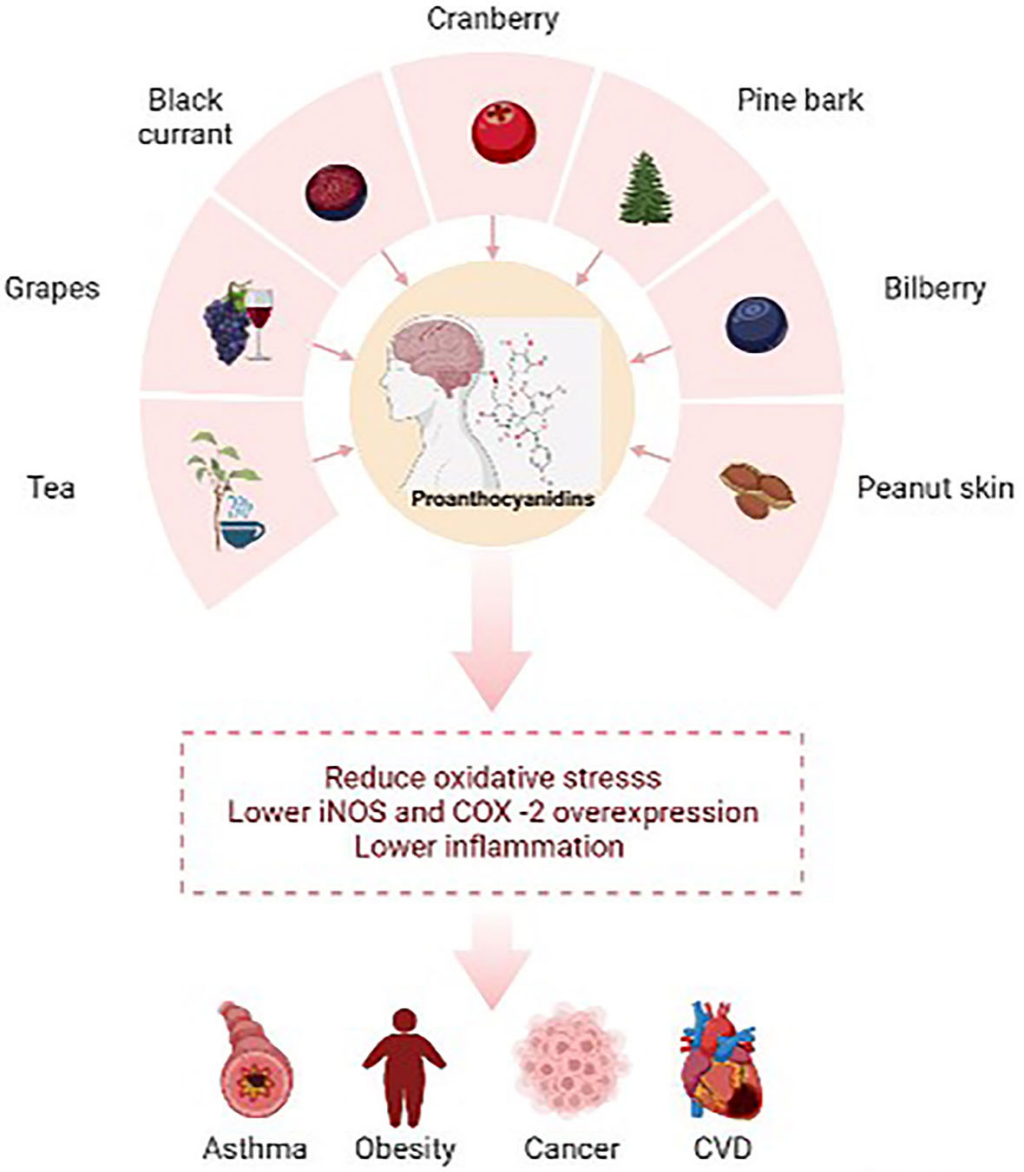
Protective mechanisms of proanthocyanidins. CVD: cardiovascular disease.

Continuous stress causes a never-ending stream of reactive oxygen species (ROS), which triggers cell death and disrupts all signaling pathways (28). However, grape seed proanthocyanidin extract (GSPE) administered to rat models in a dose-dependent manner lowered malondialdehyde (MDA), nitric oxide (NO), and calpain II protein levels while increasing the antioxidative enzyme activity (29). Additionally, it diminished the generation of lipid peroxidation, indicating anti-inflammatory, antioxidant, and neuroprotective effects. The therapeutic potentials of several proanthocyanidins are described below (30) (depicted in Figure 3).

A proanthocyanidin-rich diet can be neuroprotective and have a positive effect on other neurodegenerative diseases like Parkinson’s disease (PD), which can also be a cause of dementia. In one study, Gong et al. (31) reported the beneficial effects of proanthocyanidin on brain aging and cognitive decline induced by D-galactose. Supplementation with proanthocyanidin (30, 60, and 90 mg/kg) enhanced D-galactose-induced learning and memory impairments as well as significantly decreased brain MDA, NO, amyloid peptide, monoamine oxidase B, acetylcholinesterase (AChE), and neuronal and total NO synthase levels while increasing glutathione peroxidase and superoxide dismutase (SOD) levels. Furthermore, it prevented neuron injury in the hippocampus and suppressed P53 protein expression (32-34).

Individuals with mild, moderate, or severe AD and PD are advised to take cholinesterase inhibitors such as donepezil, rivastigmine, and galantamine (35). Additionally, the etiologic pathologies of neurofibrillary tangles (composed of p-tau) and senile plaques (Aβ) can also be targeted for potential future AD therapies. Nevertheless, it remains unclear which abnormality should be the focus of treatment to either delay or stop neurologic decline and when it should be instituted (36).

**Figure 4:**
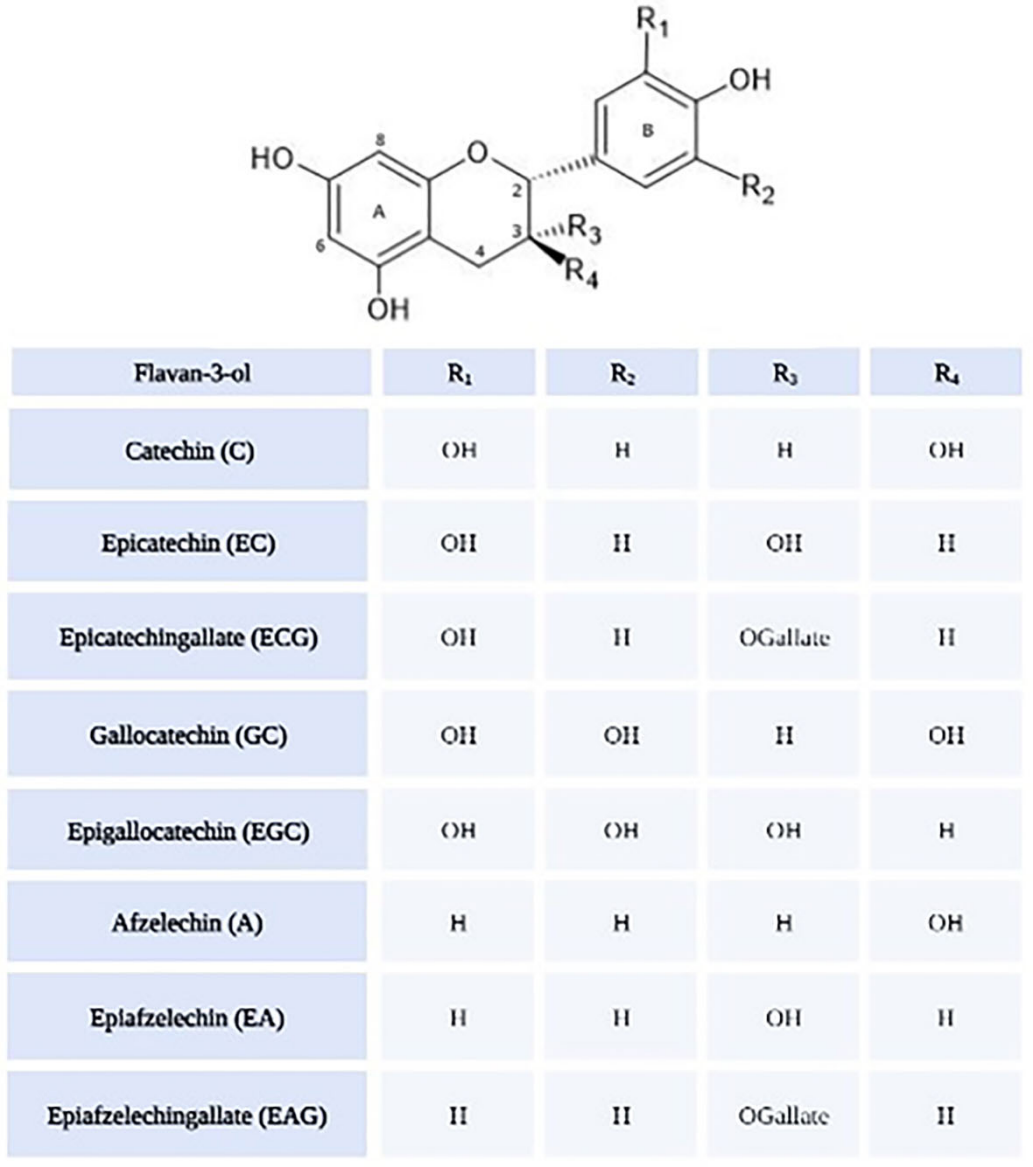
Flavan-3-ol units of the different proanthocyanidin subgroups.

Although there are many subgroups of proanthocyanidins, the procyanidin group remains the most significant one. These substances consist of condensed flavan-3-ols, which lead to the formation of oligomeric and polymeric compounds. Only three flavan-3-ols are found in procyanidins: 1) catechin (C), 2) epicatechin (EC), and 3) epicatechin gallate (ECG). Other subgroups, such as prodelphinidin or propelargonidin, consist of distinct monomeric flavan-3-ols with different hydroxylation patterns on the so-called B ring (Figure 4). The condensation of various monomeric units to oligomeric and polymeric compounds can occur via the 4-8 or 4-6 interflavonoid linkages (37).

Extensive research on animals has been conducted to determine whether the consumption of foods high in polyphenols is related to the decline in amyloid build-up and oxidative stress in AD patients. Since polyphenols have antioxidant qualities (38), they protect against neurotoxicity and influence the amyloidogenic pathway (39). Overall, the report indicates the potential protective effects of proanthocyanidin on neurodegenerative illnesses.

Numerous studies have suggested that procyanidins may improve AD-related brain neuropathology by preventing the production of toxic peptides such as amyloid precursor protein (APP) processing, amyloid-protein build-up (40), and tauopathy (41,42). Additionally, recent findings from preclinical research and *in vitro* experimental studies showed that grape seed polyphenols might interfere with specific neuropathogenic mechanisms underlying AD and suggested a potential unique function for grape seed polyphenols in treating AD (43-45). When cadmium excitotoxicity was induced in rats, proanthocyanidins (100 mg/kg a day) were seen to reverse these changes. Additionally, proanthocyanidins increased the survival rate of neuronal cells, phosphorylated Akt levels, and lowered the levels of caspase-3 (17). Improvement in spatial and object recognition impairment can increase microtubule-associated proteins (MAP)-2a and 2b levels, as well as phosphorylated neurofilament heavy subunit (PNF-H) and synaptophysin levels. However, proanthocyanidin confers neuroprotective effects in a senescence-accelerated mouse prone/8 (SAMP8) model. It also increased PNF-H levels and VGEFR-2 phosphorylation in SAMP9's brain areas (46,47).

In this systematic review, we aimed to explore the possible actions of proanthocyanidin in AD and verify if dietary supplements and therapies can improve the effectiveness of the existing armamentarium of prescribed drugs for AD. Considering the increasing number of preclinical research that supports the effectiveness of proanthocyanidin in AD and the lack of a thorough study on this subject, this review is timely. Our paper focuses on providing a comprehensive understanding of the molecular actions of proanthocyanidin in AD.

The commonly used animal models for AD induction includes intracerebroventricular (*icv*) administration of amyloid beta, *icv* injection of streptozotocin, and transgenic mouse models. Apart from these models, other preclinical non-AD models involving AD-like neuropathology such as neuroinflammation, memory impairment, reduced blood flow to the brain, oxidative stress, and mitochondrial dysfunction are also used for screening for drugs against the disease. This includes lipopolysaccharide, colchicine, scopolamine, electroshock, pentylenetetrazole, okadaic acid, olfactory bulbectomized mice (OBX), and trimethyltin (48,49). Since AD is a multifactorial disease, these models involving AD-like pathology will also play an important role in exploring drugs against the disease (50).

Other transgenic models are used in AD research, such as transgenic fruit flies (*Drosophila melanogaster*) and nematodes (*Caenorhabditis elegans*). These models offer certain advantages, such as simpler and easier to manipulate genomes, short generation times, and the ability to conduct large-scale genetic screens. However, they cannot fully replicate the complexity of human AD and are often used in conjunction with rodent models to provide complementary insights. Thus, this review only addresses the preclinical evidence involving rats and mice.

Due to the lack of a comprehensive summary of all preclinical studies on the effectiveness of proanthocyanidin, sufficient data are required to provide a more balanced experimental approach toward developing clinical studies in the future. Therefore, all relevant literature on preclinical research was critically reviewed after identifying, analyzing, and integrating the research findings to determine the credibility and significance of the data. Additionally, a nutritional strategy to treat AD is beneficial as it is affordable, simple, and safe compared to existing conventional therapeutic approaches for AD.

## Material and Methods

### Search strategy

Prior to establishing the research question, a preliminary search in PROSPERO was conducted to identify recent systematic reviews on similar subjects. Preclinical research on the effects of proanthocyanidin on AD was thoroughly peer-reviewed and searched in electronic bibliographic databases, including Scopus, Proquest, ScienceDirect, PubMed, and Google Scholar. The only search parameters used across all databases were preclinical studies and articles in English. There was no time limit in the search criteria used across all databases or in the search strategy. The literature search used a similar search term TITLE-ABS-KEY (proanthocyanidins AND Alzheimer's AND nootropic) for all databases. The search algorithm employed only terms relevant to the topic of interest and filtered unique keywords to each database.

### Inclusion criteria

The present systematic review was conducted in accordance with the Preferred Reporting Items for the Systematic Review and Meta-Analysis studies (PRISMA) procedures (51-56). This systematic review protocol was registered in the International Prospective Register of Systematic Reviews (PROSPERO) database with the code number CRD42022356301. The systematic review was conducted with the following criteria for inclusion following the population, intervention, comparison, and outcomes (PICO) methodology.

### Population

Studies involving transgenic and non-transgenic animals, including rats and mice of both genders, and investigating the nootropic effects of proanthocyanidin in AD models were included. There was no restriction on age.

### Intervention

All types of proanthocyanidin, such as natural or synthetic, rich or crude extracts, or any other varieties found in nature, were included. There were no restrictions applied to route of administration, dosage, and frequency. However, studies that investigated combination therapies with any other flavonoid or pigment were excluded.

### Comparison

This review included studies that have negative and positive controls compared to treated groups. Intervention and exposure studies were also considered.

### Study selection and data extraction

Before entering the data into Mendeley's management software, the titles, and abstracts were filtered to eliminate duplicates or unsuitable data. The second and third reviewers then double-checked the accuracy of the data. In order to eliminate any extraneous information that did not meet the requirements for inclusion, both reviewers performed a final check. The fourth and fifth reviewers clarified any discrepancies. The extraction of data from the included studies was independently conducted by the reviewers and discussed within the team.

### Risk of bias in individual studies

**Figure 5:**
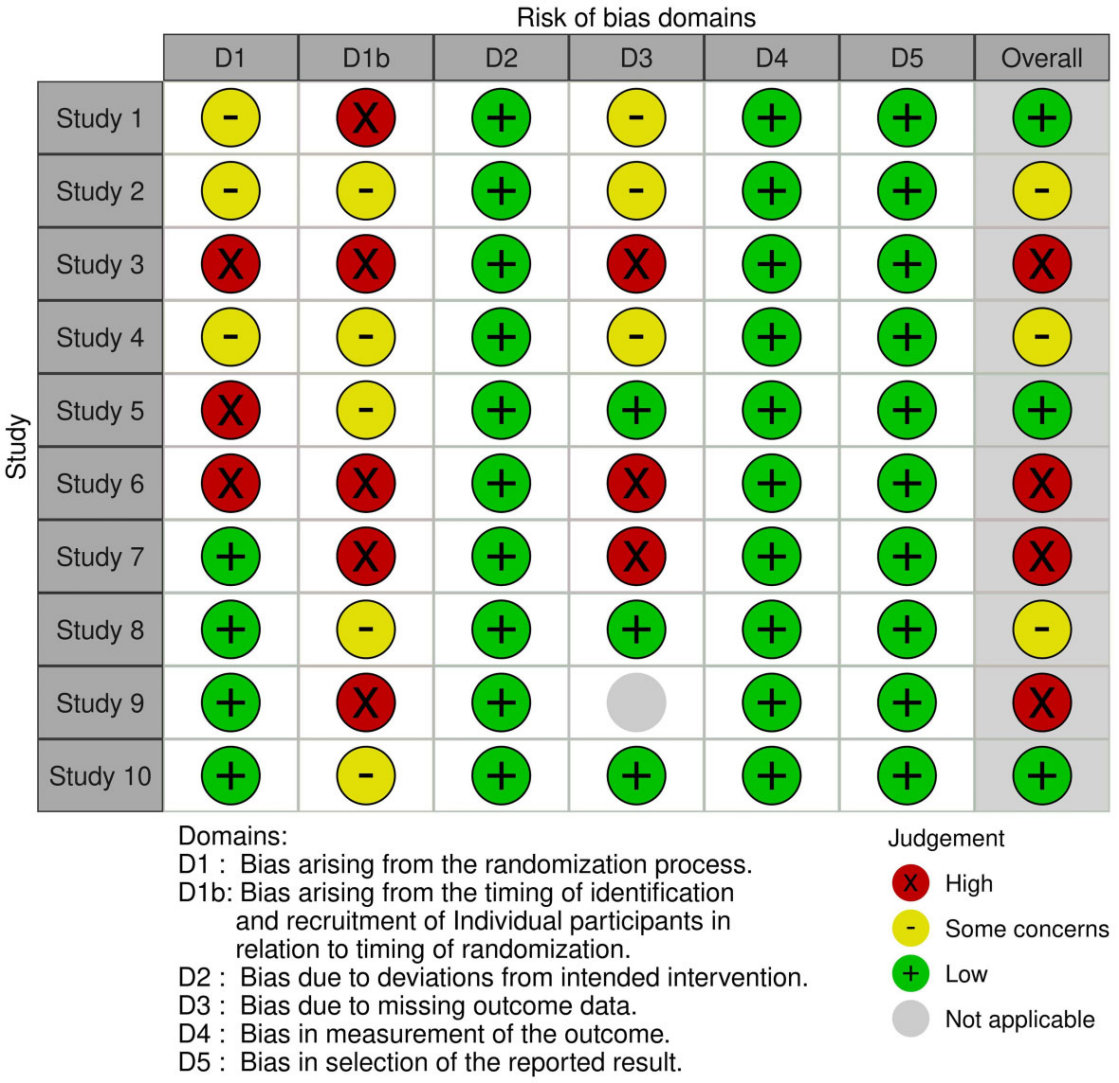
Risk of bias assessment based on the SYRCLE's Rob2 Cluster tool.

Using the Systematic Review Centre for Laboratory Animal Experimentation Risk of Bias (SYRCLE's RoB) technique, two reviewers (A.R. and A.S.) independently and critically evaluated all the reports (57). The tool comprises ten entries related to all the biases and methodological quality of preclinical investigations. Each report's risk of bias was evaluated by both reviewers, who coded as ‘yes’, ‘no’, and “unclear” for low risk, high risk, and insufficient detail to evaluate the individual risk of bias (Figure 5). Finally, any discrepancies were resolved through consultation with a third reviewer (T.T.) or by further debate until a conclusion was reached.

## Results

**Figure 6:**
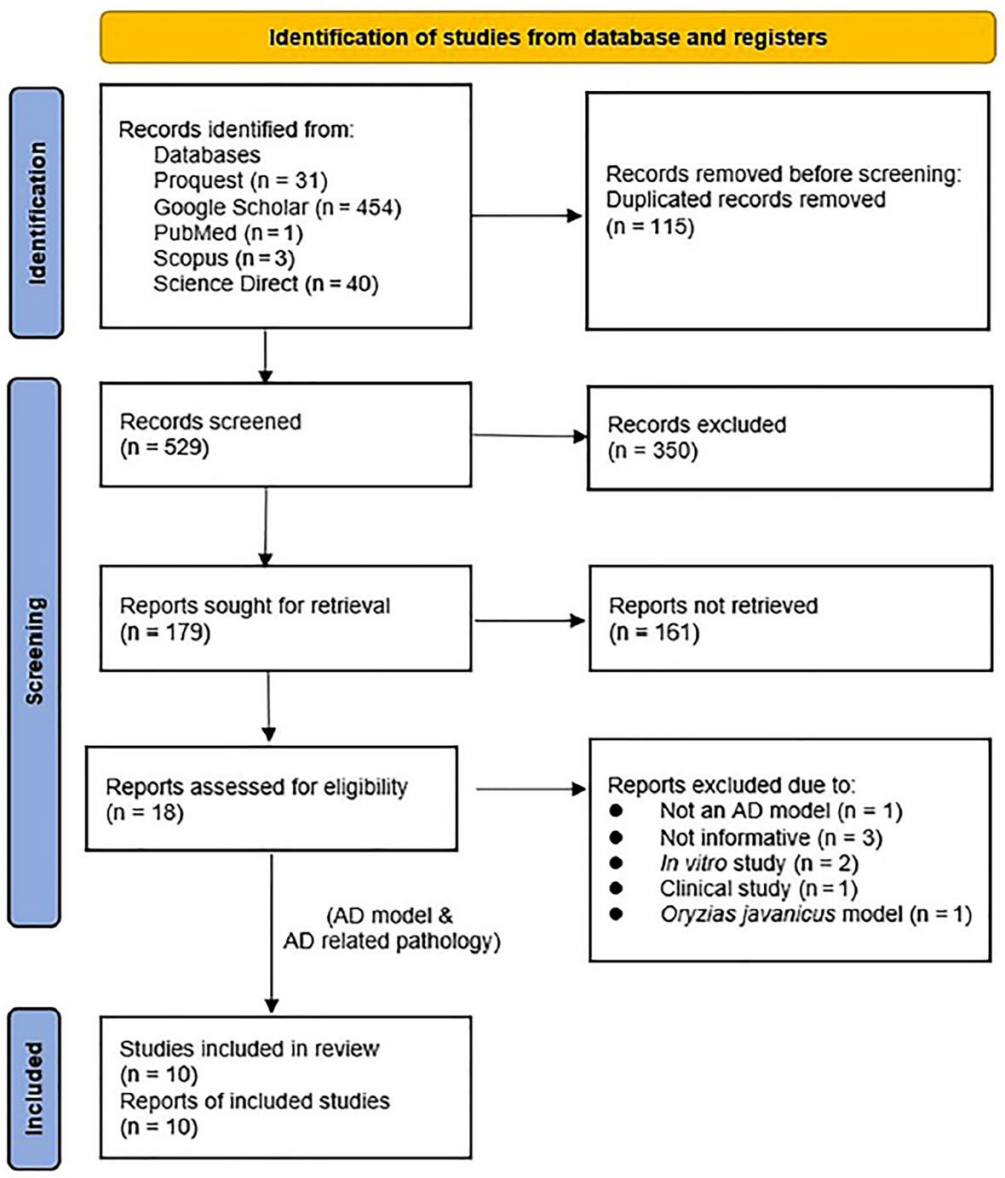
PRISMA flow chart showing the selection process (inclusion and exclusion of literature search) used in the systemic review.

A preliminary literature search retrieved 529 records across five databases. There were three from Scopus, one from PubMed, 40 from Science Direct, 31 from Pro Quest, and 454 from Google Scholar. Prior to title screening, 115 duplicate records were identified and removed, leaving only 529 records. A total of 179 reports were identified from the 350 records that were further evaluated after the initial title and abstract screening. From these numbers, only 18 were deemed eligible, while 161 reports were impossible to retrieve. Finally, data from ten reports were extracted and examined (Figure 6).

### Study characteristics

The ten studies were deemed appropriate for the systematic review with feasible statistical pooling based on eligible methodologies. The final reports included in the study were published between 2007 and 2021. One each (10%) of the ten selected research was published in 2007, 2018, 2020, and 2021, while two each (20%) were published in 2013, 2014, and 2017. Two of the included studies (20%) were from India, Iran, and Malaysia, followed by a single each (10%) from Brazil, Egypt, Vietnam, and China.

Supplementary Table S1 summarizes the data characteristics from the ten studies. Four studies used adult male Wistar rats (58-61) (n=4, 40%) and a single study utilized male Sprague-Dawley rats (62) (n=1, 10%). Four studies involved Swiss Albino male mice as AD model (40%) (63-66). A single study utilized BALB/c male mice (67) (10%). Paul et al. (58) utilized both genders of Wistar rats for their study. Martins et al. (60) utilized both adult male Wistar rats and Albino Swiss mice for their study.

Overall, the rats weighed between 180 and 450 g and mice between 20 and 50 g. In two studies, the baseline body weight of the animals was not mentioned (65,67). The age of the animals was not mentioned in four studies (40%) (58,61-63). All researchers, except one (58,61-63), utilized only male mice or rats for the experimental procedures. The study by Le et al. (65) did not state the number of animals used in each group. All ten studies in this review had more than two experimental groups.

### Characteristics of the intervention

All studies provide preliminary evidence on the effectiveness of proanthocyanidin in AD. Grape seed, *Crataegus oxycantha* L., *Trichilia catigua* A. Juss., *Prunus domestica* L., *Elaeis guineensis* Jacq., *Ocimum sanctum* L., and *Trachyspermum ammi* (L.) Sprague. are plants that naturally contain proanthocyanidin. A combination of natural sources of proanthocyanidin with the aerial parts of *Markhamia platycalyx* (Baker) Sprague., *Camellia sinensis* (L.) Kuntze., and stalks of *Schotia brachypetala* Sond. were used in the study by Hassaan et al. (63) (10%). Three studies (59,61,62) used grape seed extract (GSE) as an intervention (30%). A single study (74) utilized the powder of seeds from *Trachyspermum ammi* (L.) Sprague. as an intervention in order to explore its beneficial effects on learning and memory of mice (10%). Finally, nine out of ten preclinical studies used extracts (58-65,67)

## Discussion

Nootropics are substances that have a positive impact on cognitive processes. These drugs improve memory and learning while reducing the impairment in cognitive abilities caused by diseases and brain trauma. Hypoxia, cerebral ischemia, and amnesia-inducing agents interfere with learning and retention in passive avoidance, positive reinforcement, and learning paradigms. The effects of several nootropic drugs have been investigated in animal models of compromised cognitive capabilities.

This systematic review indicated current novel evidence of nootropic effects of proanthocyanidins, which can ameliorate AD, thus highlighting the value of existing literature as a foundation for future research studies. The systemic evaluation of *in vivo* preclinical trials confirms the efficacy of proanthocyanidins as a therapeutic option in treating AD. Some animal models that can indirectly lead to AD-like pathology were also taken into consideration in this review, as these studies highlighted the multifactorial role of proanthocyanidin in combating AD and similar diseases.

All but one study (59) did not clearly indicate the age of the animals used. This is a major bias based on SYRCLE's risk of bias tool, which was used by the authors for scoring the papers.

First, the studies consistently demonstrated the potential of proanthocyanidin to improve cognitive function in animal models of AD. The positive effects were observed across various cognitive domains, including memory, learning, attention, and motor function. The antioxidant properties of proanthocyanidin were frequently implicated as a mechanism underlying the neurocognitive benefits. Oxidative stress and the resulting damage to neuronal cells are known to play a significant role in the pathogenesis of AD. The ability of proanthocyanidin to scavenge free radicals and reduce oxidative stress markers suggests its potential as a therapeutic agent in combating the neurodegenerative processes associated with the disease.

Furthermore, the studies highlighted the modulation of cholinergic neurotransmission as another potential mechanism by which proanthocyanidin exerts its neurocognitive effects. AD is characterized by a deficiency in acetylcholine, a neurotransmitter critical for memory and cognitive functions. The studies indicated that proanthocyanidin can inhibit acetylcholinesterase, the enzyme responsible for the breakdown of acetylcholine, thereby increasing its concentration and enhancing cholinergic transmission.

There were no restrictions in any database and all the included reports were published since 2007, thus indicating that the potential of proanthocyanidins in treating AD has started to gain increasing attention over time, especially in the last 15 years. A significant part of the research in this area was published between 2013 and 2017. Studies that measured outcomes other than those related to neurodegeneration, AD, or dementia were excluded. The studies were unrestricted by year, and only original studies in English were included. Clinical studies were excluded and *in silico* and *in vitro* studies were included. Studies that had any of the following outcome indicators were included: behavioral and biochemical parameters like acetylcholinesterase concentrations, butyrylcholinesterase levels, amyloid-beta, NFTs, neuro-protective markers, oxidative markers, microarray studies, histopathology of the brain, real-time qRT-PCR studies, extracellular signal-regulated protein kinase (ERK), protein kinase B (PKB/Akt), brain-derived neurotrophic factor (BDNF), total phenolic content, GSH assay, western blot, and any other neurodegenerative markers. Studies were conducted by researchers from India, Iran, Malaysia, Vietnam, China, Brazil, and Egypt to consider Oriental medicine as a therapeutic option in AD.

Most of the studies were published in Asian countries. However, based on the World Health Rankings 2022 (WHR), United Kingdom, Nordic regions like Iceland and Finland, Albania in south-eastern Europe's Balkan peninsula, and Slovakia in central Europe have the top five AD and dementia death rate. Due to the inconsistent nature of the data from the female gender, perhaps due to hormonal shifts or the menstrual cycle, all ten included studies utilized male rodents exclusively. However, a single study by Paul et al. (58) performed the investigation on either sex. Two studies (58,64) used scopolamine (1 mg/kg) as the AD inducer model. The other studies utilized inducers like pentylene tetrazole (PTZ) (35 mg/kg/day), lipopolysaccharides (LPS) (0.8 mg/kg), and an electroshock (20V). Leow et al. (67) used transgenic BALB/c male mice.

Sarkaki et al. (59) demonstrated that GSE exhibited remarkable cognitive-enhancing activity since it can improve spatial memory and prevent memory deficits in the central nervous system (CNS). Based on the report by Yamakoshi et al. (47), GSE is non-toxic to rats. GSE also improved the antioxidant status and reduced lipid peroxidation caused by free radicals in the CNS of aged rats (68). It can inhibit cell death signaling mediated by JNK-1 and c-JUN, as well as other pro-apoptotic transcription factors and genes. Additionally, it can scavenge oxygen-derived free radicals and prevent lipid peroxidation as well as having anti-inflammatory properties. Moreover, GSE can reduce the production of pro-inflammatory cytokines (69).

Paul et al. (58) investigated the effectiveness of *Crataegus oxycantha* L. methanolic extract against dementia and found that it significantly reduced the level of oxidative stress markers in the brain of scopolamine-treated rats. The investigators also reported enhanced learning and memory activity in the rats, which occur via 1) reduction of the rat's transfer latency time on the elevated maze, 2) reduction in acetylcholine esterase activity, and 3) increased SOD levels. The cognitive behavior of the animal is influenced by a variety of mediators including noradrenaline, acetylcholine, dopamine (DA), serotonin (5-HT), GABA, glutamate, nitric oxide, and peptides (70). The neuroprotective activity of *Trichilia catigua* A. Juss. allows regulation of the antioxidant levels of DPPH and AChE due to the presence of proanthocyanidin (60).

In another study, two phenolic-rich extracts of *Schotia brachypetala* Sond and *Markhamia platycalyx* (Baker) Sprague were investigated (63). Compared to untreated animals, the administration of the two extracts altered energy metabolism, signaling pathways, and gene expression involved in the ability of neurons to strengthen and change synaptic connections. Overall, the findings indicated that polyphenols impact mental health and cognition. Polyphenols have been linked to increased BDNF expression in addition to their anti-inflammatory and antioxidant properties and help to reverse neuronal atrophy and behavioral abnormalities. BDNF is a neurotrophic agent renowned for its impact on the maintenance, survival, growth, and differentiation of neurons (71). Furthermore, the animals that received *ip* injections of *Schotia brachypetala* Sond and *Markhamia platycalyx* (Baker) Sprague. phenolic extracts had significantly lower levels of amyloid beta-42 (Aβ42) compared to mice administered with LPS.

In this review, it was hypothesized that proanthocyanidins can reduce the symptoms of AD. The extracts of grape seed, *Crataegus oxycantha* L., *Trichilia catigua* A. Juss., *Prunus domestica* L., *Elaeis guineensis* Jacq., *Ocimum sanctum* L., *Trachyspermum ammi* (L.) Sprague., and other naturally occurring sources of proanthocyanidins have been utilized as therapeutic interventions for AD in the ten studies reviewed. All papers demonstrated the therapeutic effectiveness of proanthocyanidins against various variables and mechanisms that may promote both the initiation and advancement of AD. This diversity suggests that proanthocyanidin's neurocognitive benefits may extend beyond a specific source, indicating the broader applicability of this compound in AD treatment. Nevertheless, the dose and duration of therapy varied widely across the different studies based on the research objective, method of administration, rodent model type, and intervention used.

Flavan-3-ols procyanidin B isomers (Sb3.Sbs) extracts have been shown to be highly effective in treating degenerative conditions associated with oxidative stress such as AD, as seen with *Schotia brachypetala* Sond. in this study. The finding was further confirmed in a study where the cerebellum, cerebral cortex, and hippocampus tissues of rats were confirmed to be protected from lipid and protein oxidative damage (72). Procyanidins also significantly reduced Aβ42 aggregation and had a dose-dependent ability to disintegrate Aβ42 aggregates (41). Additionally, the treatment reduced the Aβ42 load in the treated mice compared to untreated LPS-injected mice, as confirmed by ELISA. Moreover, procyanidin isomers, daidzein, procyanidin dimer gallate, naringin, ellagic acid, quercetin 3-o-glucuronide, quercetin hexose gallic acid, quercetin hexose protocatechuic acid, and quercetin 3-O-rhamnoside were identified (71).

Proanthocyanidin, one of the active components of GSE, has been demonstrated in some studies to prevent glutamate-induced cell death in cultured rat hippocampus neurons by inhibiting calcium signals and by producing nitric oxide (68). Additionally, Sarkaki et al. (61) confirmed the potential benefits of GSE. The benefits may be due in part to its antioxidant properties and its antagonist effects on brain glutamate activity.

The findings by Singh et al. (64) showed that *Prunus domestica* L. fruit extract (EEPD) has anti-inflammatory and anti-amnesic effects on scopolamine-induced amnesia. The effects are closely related to a better behavioral performance and a more controlled cholinergic transmission that occurs via suppression of the cholinesterase enzyme. Furthermore, *P. domestica* L. affected the cholinergic neurotransmission and impacted habituation and behavioral memory, as confirmed by the Y-maze and open-field habituation memory test. There were promising neuroprotective outcomes and cognitive enhancement after scopolamine-induced amnesia, where EEPD suppressed the AChE enzyme in mice brain at two doses (200 and 400 mg/kg). The findings indicate the neurocognitive benefit exerted by the *P. domestica* L. extract.

In the study conducted by Leow et al. (67), three isomers of caffeoyl shikimic, caffeic, protocatechuic, and hydroxybenzoic acids were confirmed as the primary components of oil palm phenolics (OPP). When mice were administered with OPP, the cognitive and motor functions were improved, indicating its neuroprotective effect on upregulated neurotrophic genes. A hallmark lesion of AD, intracellular NFTs, are thought to be broken down by tyrosine phosphatases (Ptprn and Ptprt), whereas the ionotropic glutamate receptor (Gria3) is crucial for synaptogenesis and the neuronal circuitry (73). Inflammation has also been associated with brain aging. Therefore, it is noteworthy that inflammation-related genes such as secreted phosphoprotein 1 or osteopontin (Spp1), serum amyloid A3 (Saa3), and apolipoprotein D were down-regulated by OPP supplementation in the study.

Le et al. (65) reported that *Ocimum sanctum* Linn (OS) has an ameliorative effect on OBX-induced cognitive impairments in mice, as mediated by the hippocampal neurogenesis, which occur via stimulation of central cholinergic systems and restoration of the expression of VEGF. Administration of OS extract and donepezil restored the OBX hippocampus downregulated expression of VEGF. VEGF is a key signaling molecule that promotes endothelial cell proliferation, motility, and resistance to apoptosis. VEGF and its receptors (including VEGF receptor-2 or VEGFR2) are found throughout the CNS. They are located mainly in neurons, choroid plexus epithelial cells, neural progenitor cells, and astrocytes (74,75).

Soni et al. (66) reported that treatment with TASP (*Trachyspermum ammi* seed powder) attenuated oxidative stress as indicated by brain MDA and nitrite levels, as well as amelioration of brain glutathione levels. Their findings confirmed that the antioxidant properties of TASP are responsible for its anti-amnesic and memory-enhancing properties in mice. Administration of TASP also reduced brain nitrite levels and protected against oxidative damage caused by nitrite. Because of the AChE inhibitory action, the use of ajowan seeds in the daily diet is recommended as it may benefit AD patients (66).

Based on a western blot analysis, Zhen et al. (62) revealed that seizures caused by pentylene tetrazole (PTZ) substantially increased hippocampus caspase-3 activation and cytosolic caspase-3 and cytochrome c release. Moreover, Nissl staining revealed considerable pyramidal cell loss with ultrastructural evidence of apoptosis. Additionally, the electron imaging revealed that mitochondria in the PTZ group were damaged. Nevertheless, pre-treatment with GSPE restored all the morphological alterations. It was concluded that treatment with mitochondrial bioenergetics ameliorate oxidative stress by using GSPE and non-pharmacological treatments, and is effective for epilepsy control.

However, it is important to acknowledge the limitations of the reviewed studies. All the studies were conducted on animal models, and the translation of findings to human patients may differ. The variation in dosage, administration routes, and treatment duration also limited the ability to draw definitive conclusions about optimal therapeutic strategies.

## Conclusion

This review thus provides promising evidence for the neurocognitive effects of proanthocyanidin in AD. Research involving animal models have confirmed that proanthocyanidins offer considerable health advantages. The antioxidant properties, cholinergic modulation, and broad applicability across different sources suggest its potential as a therapeutic intervention. However, further research is required to bridge the gap between preclinical evidence and clinical application. Additionally, investigating potential synergistic effects with other therapeutic approaches and assessing long-term effects will contribute to a more comprehensive understanding of the potential of proanthocyanidin as a neurocognitive enhancer in AD.

## Future perspectives

**Figure 7:**
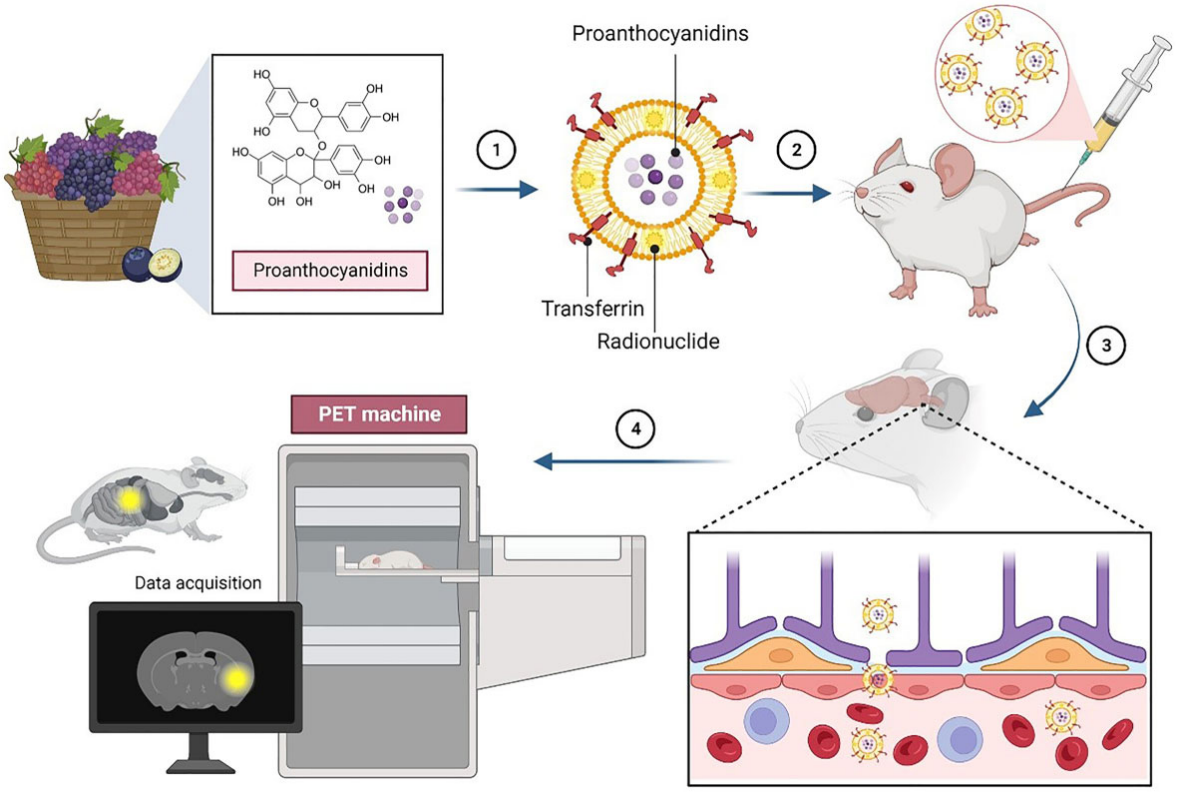
Future perspective of liposomal proanthocyanidins for the management of AD. PET: positron emission tomography machine.

Large double-blind clinical trials on proanthocyanidins are required to confirm the clinical efficacy and safety, which will allow its clinical application as an effective nootropic agent against AD. As for future perspectives, the use of nanotechnology such as liposomes can be employed to encapsulate proanthocyanidins to further enhance the safety and bioavailability of the bioactive compounds. With the help of conjugated transferrin, the formulation can be targeted toward the brain, transporting the molecules across the blood-brain barrier (BBB). Liposomes can be further exploited by including radionuclides in the lipid bilayer for diagnostic imaging and determining biodistribution (Figure 7).

The effectiveness and safety of proanthocyanidin in human populations must be established by additional studies, including well-planned clinical trials. To develop preclinical discoveries into useful treatment strategies for people with AD, more analysis and clinical studies are required.

## Supplementary Material

Click here to view [pdf].
