# Supplementary material for: Neurocognitive effects of proanthocyanidin in Alzheimer's disease: a systematic review of preclinical evidence
Source: Braz J Med Biol Res. 2024 Nov 4;57:e13587. doi: 10.1590/1414-431X2024e13587 (PMC11540257; doi:10.1590/1414-431X2024e13587)
Supplement: Supplementary file 1 [file 1414-431X-bjmbr-57-e13587-suppl.pdf]

Table S1. Characteristics of the ten included reports.

Author/Country	Animal/strain/ weight/age	Induction model	Sample size/groups	Source of proanthocyanidin	Dose and route	Duration	Outcome measures		
							Biochemical parameters	Behavioral tests	Conclusions
Sarkaki et al. (59) Iran	Aged male Albino Wistar rats; Body weight >300 g; 2 years old	–	15 animals/group<br>Two groups;<br>I-Vehicle treated Control<br>II-Treated GS	Grape seed extract	100 mg/kg; oral gavage	30 days	–	1. MWM	Results demonstrated that the water maze performance improved due to the antioxidant properties of the polyphenols present in GSE. The treated groups showed ↓ escape latency time, ↑ swimming speed and ↑ cognitive activity.
Paul et al. (58) India	Wistar rats either sex	Scopolamine (1 mg/kg)	6 animals/group<br>6 groups;<br>I-Vehicle control<br>II-Negative control (Scopalamine)<br>III-Standard control (Piracetam)<br>IV-received PECO orally<br>V-Administered with CHCO<br>VI-Administered with MECO	Leaves of *Crataegus oxyacantha* L. (Hawthorn)	200 mg/kg; oral gavage	15 days	1. AChE assay<br>2. Lipid peroxidase<br>3. SOD assay	1. Elevated plus maze model<br>2. Monoamine-mediated behavior	*Crataegus oxyacantha* L. caused ↓ AChE activity, ↑ SOD concentration and restored the perturbed oxidative stress status in the brain. It decreased the number of head twitches and the duration of catalepsy. The results presented a trending therapeutic strategy for prevention of dementia by enhancing deficits in learning and memory and preventing oxidative damage.
Martins et al. (60) Brazil	Male albino Swiss mice and male Wistar rats		Randomly-divided into the different groups (n=8–10)	Extracts of the barks of *Trichilia catigua* A. Juss. (catuaba)	Gavage (oral administration, *po*) 0.1 mL per 10 g body weight (mice) or 0.1 mL/100 g (rats) (50 and 300 mg/kg, *po*) for 21 days. Hydroalcoholic extract (25 and 250 mg/kg, *po*) for 14 days	14 days	1. DPPH assay<br>2. AChE activity	1. Forced treadmill exercise<br>2. Motor activity<br>3. Stress by immobilization and cold stressor<br>4. Classic fear conditioning	Cinchonains and procyanidins in *T. catigua* A. Juss. was the best antioxidant and anticholinesterase for the hydroalcoholic extract. Inhibition of AChE caused ↑ concentration and time of acetylcholine on synaptic cleft, thus facilitating the cholinergic transmission.
Hassaan et al. (63) Egypt	Adult male Swiss albino mice	Lipopolysaccharide (0.8 mg/kg), *ip*	Groups of 8–10 mice	Extract of aerial parts of *Markhamia platycalyx* (Baker) Sprague, *Camellia sinensis* (L.) Kuntze.and stalks of *Schotia brachypetala*.	100 mg/kg/day; oral gavage	6 days	1. ELISA<br>2. Measurement of Aß 42	1. Y-maze<br>2. Object recognition	The findings demonstrated the strong potential of phenolic-rich extracts of *Schotia brachypetala, Camellia sinesis* (L.) Kuntze., *Markhamia platycalyzx* (Baker) Sprague, and piceatannol, which are therapeutic leads against AD. When compared to animals that received LPS injections without treatment, the drugs also ↓ Aß 42 load considerably, as confirmed by ELISA.

Sarkaki et al. (61) Iran	Adult male Wistar rats	Common carotid arteries occlusion (2CCAO)	8 animals/group<br>4 groups:<br>I-Sham+Veh<br>II-Isch+Veh<br>III-Sham+GSE<br>IV-Isch+GSE	Grape seed extract	100 mg/kg; oral gavage	28 days	1. Glutamate activity<br>2. Electrophysiology	1. Passive avoidance memory<br>2. STL<br>3. Sensory motor activities	The potential benefits of GSE may be due (in part) to its 1) antioxidant properties and 2) antagonistic effects on brain glutamate activity. In rats with 2CCAO, GSE therapy markedly ↓ memory loss and boosted hippocampus LTP.
Singh and Manan (64) Malaysia	Swiss male mice	Scopolamine (1 mg/kg)	6 animals/group<br>4 groups:<br>I-(Control)<br>II-(Scopolamine)<br>III-(Scopolamine + 200 mg/kg PDEE)<br>IV-(Scopolamine + 400 mg/kg PDEE)	*Prunus domestica* L. fruit extract (EEPD)	200 and 400 mg/ kg	15 days	AChE enzyme<br>1. Total phenolic content<br>2. Free radical scavenging ability	1. Y-maze test,<br>2. Open-field test<br>3. Traction test	The findings revealed that EEPD has a significant ameliorating impact on AChE inhibition on scopolamine-induced amnesia and is effective in treating memory and learning impairment.
Leow et al. (67) Malaysia	Male BALB/c mice	–	5 animals/group<br>2 groups;<br>I-Control<br>II-Treatment group	Palm oil phenolics (OPP) (*Elaeis guineensis* Jacq.)	2.62 mL/day	6 weeks	1. Total RNA<br>2. qRT-PCR studies on *Fos* and *Bcarl* ERK<br>3. PKB/Akt<br>4. CREB<br>5. BDNF	1. Water maze<br>2. Rotarod	Microarray examination of the mouse's brain tissue revealed that when OPP was administered to mice that received a regular diet, 113 genes were upregulated while 145 genes were significantly downregulated compared to the controls. It was concluded that OPP's neuroprotective and anti-inflammatory properties contributed to the enhancement of mouse cognitive and motor functions.
Le et al. (65) Vietnam	Male *Swiss albino* mice	Olfactory bulbectomized mice (OBX)	Two groups<br>I-Sham group<br>II-OBX group	*Ocimum sanctum* L., extract	200, 400 mg/kg; oral gavage	1 week	1. VEGF<br>2. VEGFR2	1. Modified Y maze test<br>2. Novel object recognition test	The levels of OBX-induced VEGF gene and protein expression were reduced. However, treatment with OS and DNP reversed the effect without affecting the expression of the VEGFR2 gene. The results revealed that OS treatment can reduce the cognitive deficits and neurohistological damage produced by OBX and that these benefits are mediated by central cholinergic system stimulation and VEGF production.
Soni and Parle (66) India	Male Swiss mice	Electroshock (20 V, AC)	5 equal groups<br>6 animals/group<br>I-Control<br>II-Positive control<br>III to VI-TASP	*Trachyspermum ammi* (L.) Sprague. Seed powder (TASP)	0.5, 1.0, and 2% w/w; oral gavage	10 days	1. Brain AChE<br>2. Serum total cholesterol<br>3. Brain MDA<br>4. Thiobarbituric acid<br>5. Brain Rreduced GSH level	1. PAP<br>2. ORT<br>3. Locomotor function	The acetylcholinesterase enzyme in the brain was blocked by TASP, which increased acetylcholine concentration in the brain homogenate and decreased memory. The

							6. Brain nitrite Level<br>7. Serum cholesterol		memory performance parameters overall indicated significant improvement. ↓ brain's MDA and nitrite levels as well as restoration of the decreased brain GSH levels minimized oxidative stress.
Zhen et al. (62)<br>China	Male Sprague-Dawley rats	Pentylenetetrazole (PTZ),<br>35 mg/kg/day (*ip*)	5 groups:<br>I-Control group<br>II-PTZ group<br>III, IV-PTZ + GSPE<br>V-GSPE alone group	Grape seed proanthocyanidin extract	35 mg/kg/day	36 days	1. Hippocampal MDA<br>2. GSH levels<br>3. Pro-apoptotic factors Cyt *c*, caspase-9 and caspase-3	1. MWM	By safeguarding mitochondrial function and reducing caspase-3-dependent apoptosis, pre-treatment with GSPE reduced further cognitive deterioration. Additionally, it reduced oxidative stress in the brain of rats suffering from recurrent seizures.

GSE: Grape seed extract; MWM: Morris water maze; PECO: Pet. ether extract of *Crataegus oxyacantha*; CHCO: Chloroform extract of *Crataegus oxyacantha*; MECO: Methanol extract of *Crataegus oxyacantha*; AChE: Aetylcholinesterase; Ach: Acetylcholine; SOD: Superoxide dismutase; *po*: by mouth; DPPH: y 2,2-diphenyl-1-picryl hydrazyl; ELISA: Enzyme-linked immunoassay; Aß: Amyloid beta; LPS: Lipopolysaccharide; STL: Step-down latency; 2CCAO: Common carotid arteries occlusion; LTP: Long term potentiation; PDEE: *Prunus domestica* L. ethanolic extract; EEPD: Ethanolic extract *Prunus domestica* L.; OPP: Oil palm phenolics; RNA: Ribonucleic acid, qRTPCR: Quantitative reverse transcription-polymerase chain reaction, ERK: Extracellular signal-regulated protein kinase; CREB: Cyclic adenosine monophosphate-response element-binding protein; BDNF: Brain-derived neurotrophic factor; OBX: Olfactory bulbectomized; VEGF: Vascular endothelial growth factor; OS: *Ocimum sanctum* L.; VEGFR2: VEGF receptor type 2; DNP: Donepezil; TASP: *Trachyspermum ammi* (L.) sprague seeds powder; MDA: Malondialdehyde; ORT: Object recognition task, GSH: Glutathione, PAP: Passive avoidance paradigm, PTZ: Pentylene tetrazole, *ip*: Intra-peritoneal; Cyt c: Cytochrome c; GSPE: Grape Seed Proanthocyanidin Extract; DNP: Donepezil.
